# Audit on the Adequacy of Consenting Practices for Neck of Femur Fracture Surgeries

**DOI:** 10.7759/cureus.48565

**Published:** 2023-11-09

**Authors:** Hector Perera, Adedoyin M Wusu, Alhashash Mohammad, Mahdi Z Qulaghassi, Adekunle Adekola, Madhu Rao

**Affiliations:** 1 General Surgery, Royal Sussex County Hospital, Brighton, GBR; 2 Trauma and Orthopaedics, St. Richard’s Hospital, Chichester, GBR; 3 Trauma and Orthopaedics, Medway Maritime Hospital, Gillingham, GBR; 4 General and Colorectal Surgery, St. Richard’s Hospital, Chichester, GBR

**Keywords:** orthopaedics, postoperative complications, audit, informed consent, femoral neck fracture

## Abstract

Introduction: Doctors are bound to obtain informed written consent prior to any form of surgical procedure on a patient. The General Medical Council (GMC) and the Royal College of Surgeons of England (RCS) provide guidance on what constitutes valid consent. Failure to obtain valid and adequate consent can have legal ramifications. All relevant material risks associated with the surgery must be discussed with the patient during the consenting process.

Materials and methods: This was a retrospective cross-sectional study of the consenting practices for neck of femur fracture surgeries, covering a period of three months, from the 15th of April to the 15th of July 2023. We evaluated the consent forms of 100 patients, of which 63 were consent form-1 and were included in the study. The British Orthopaedics Association (BOA)-endorsed consent forms, together with the RCS and GMC guidance on consent, which were used as the standard for the audit.

Results: The majority of the consents were obtained by senior house officers (SHO) and core surgical trainees who did not have prior formal orthopaedic training (52.4%). The risks that were most frequently documented were infection, blood clots (deep vein thrombosis and pulmonary embolism), and bleeding, with documentation rates of over 90%. Prosthetic joint dislocation following hemiarthroplasty or total hip replacement was not mentioned in 22.2% of the forms. Neurovascular injury was not documented in 20.6% of the consent forms. Less than 75% documentation rates were observed for postoperative pain (74.6%), anesthetic complications (73%), failure (malunion/non-union/loosening of prosthesis) (68.3%), leg length discrepancy (60.3%), bone damage/fracture (50.8%), death (49.2%), wound-related complications/scars (42.9%), and hip stiffness (14.3%). None of the patients had been advised about the probable need for catheterization following surgery. We also noted that 22.2% (n=14) of the consent forms did not contain the diagnosis or the indication for surgery, 12.7% (n=8) did not mention the intended benefits, and 28.6% (n=18) of the consent forms had no mention of the responsible consultant. We also noted that in 25.4% (n=16) of the cases, the possible requirement of a blood transfusion had not been mentioned.

Conclusion: The audit revealed several deficiencies in the consenting of patients for neck and femur fracture surgeries. There were poor documentation rates for risks associated with surgery, especially the less common and rare ones. We also identified several deficiencies in the remaining aspects of the consent forms that were not in keeping with the GMC and RCS guidance on consent. The lack of orthopaedic training and knowledge among the senior house officers and core trainees may be a contributing factor.

## Introduction

Doctors are bound both legally and ethically to obtain informed written consent before any form of surgical procedure on a patient. It is the responsibility of the doctor to openly disclose all aspects of the procedure, including the intended outcomes, the probable complications and risks, and alternative options available, including the option of no treatment or action, so that the patient has adequate information to make an informed decision about their life and to exercise their bodily autonomy. It is important that patients are involved in the decision-making process and are provided an opportunity to express their concerns and queries. Both the General Medical Council (GMC) and the Royal College of Surgeons of England (RCS) provide comprehensive guidance on what constitutes valid consent. For consent to be valid, the patient must possess the capacity to decide, voluntarily provide consent, and be provided with adequate and appropriate information to make the decision. From a legal standpoint, not only is it important to have a comprehensive discussion with the patient on the planned procedure, but it is also imperative that said information be put down in writing [1,2].

The website, www.orthoconsent.com, is a free online platform that provides orthopaedic surgeons with access to comprehensive and well-structured consent forms for a variety of orthopaedic procedures and has been endorsed by the British Orthopaedics Association (BOA). These forms have been developed in line with the Department of Health guidelines and with strict legal and ethical scrutiny [3,4]. These consent forms clearly outline the planned procedure, the indication for surgery, the intended benefits, and probable risks, along with their relative rates. They also contain the patient identification details at the top of the form, together with the name of the responsible surgeon. The patient’s signature, name, date of signing, and the consenting doctor’s name, designation, signature, and date of signing are denoted at the bottom of the form. The hip fracture consent form on the website was taken as the standard against which the consent forms in our study were assessed.

St. Richard’s Hospital is a District General Hospital in Chichester, United Kingdom that provides care to residents in the Southwest Sussex and East Hampshire regions. The trauma and orthopaedics department carries out a daily trauma surgical list in addition to a large volume of elective orthopaedic surgeries. A neck-femur fracture is a common presentation encountered among the elderly. It has been shown that early intervention within 48 hours has significantly lower one-year mortality as opposed to delayed intervention [5,6]. The type of surgery carried out depends on the nature of the fracture and other patient-related factors. The common interventions that are carried out in our unit for hip fractures are hemiarthroplasty, total hip replacement, and internal fixation with a dynamic hip screw or intramedullary nail. Consenting for surgeries for neck and femur fractures is usually done by the registrars, senior house officers, and core trainees. The aim of this audit was to determine the adequacy of the consenting practices for neck and femur fracture surgeries. The objectives were to determine the relative rates of documentation of risks associated with surgery and the overall completeness of the consent forms in accordance with GMC and RCS guidance. 

## Materials and methods

This audit was a retrospective cross-sectional study of the consenting practices for neck and femur fracture surgeries, covering a period of three months, from the 15th of April to the 15th of July, 2023. We used a consecutive sampling technique and analyzed the consent forms of 100 patients who were diagnosed with femoral neck fractures and subsequently underwent surgery on the trauma list during the study period. Out of the 100 consent forms that were evaluated, 37 were consent form-4, which meant that they did not have the capacity to consent (Abbreviated Mental Test/AMT Score of <8). These consent forms were excluded from the study. A total of 63 were consent form-1 and, therefore, were included and analysed in this study.

The data regarding the number of patients admitted with neck or femur fractures and those that underwent surgery during the audit time frame were obtained from ‘e-trauma’, which is an online database used by the trauma and orthopaedic department. All patient documents in the trust, including consent forms, are uploaded after patient discharge to ‘Affinity’, which is the secure online platform used at St. Richard's hospital. Hence, all the consent forms were easily accessed through this system. As this was carried out as an audit within the orthopaedic department and was a retrospective analysis of existing documents, ethical clearance was not required. The audit was registered with the clinical audit department of the trust.

As the standard for our audit, we used the General Medical Council Guidance on Professional Standards and Ethics for Doctors: Decision Making and Consent (2020), the Royal College of Surgeons of England Consent: Supported Decision-Making (2016), and the model consent form for hip fracture surgeries on the British Orthopaedic Association-endorsed website, www.orthoconsent.com [1,2,4]. The consent forms were then analyzed individually to see if the overall documentation was complete and in compliance with the GMC and RCS guidance. We assessed the documentation of the following information: patient details, responsible consultant, diagnosis and title of the surgery, intended benefits, probable risks, type of anaesthesia, the probable need for blood transfusion, consenting doctor's name, designation, and signature, and the patient's signature and name. Special emphasis was given to the rate of documentation of the risks associated with the procedure. The statistical analyses were carried out using IBM Corp. Released 2021. IBM SPSS Statistics for Windows, Version 28.0. Armonk, NY: IBM Corp, with statistical significance defined as P<0.05.

## Results

From the 63 consent forms for neck and femur fracture surgeries that were included in the study, 49.2% (n=31) underwent hemiarthroplasty of the hip, 42.9% (n=27) underwent surgical fixation with either a dynamic hip screw or an intra-medullary nail, and finally 7.9% (n=5) underwent total hip replacement. In 52.4% (n=33) of the cases, consent for the surgery was obtained by a senior house officer (SHO) or core trainee who had no prior formal orthopaedic training. This was followed by specialty registrars, who had taken 46.0% (n=29) of the consents. Only one consent was obtained by a consultant (Table 1). 

**Table 1:** Summary of the type of surgery carried out for the neck of femur fractures and the person taking consent

Type of Surgery	Number (n)	Percentage (%)
Hemiarthroplasty	31	49.2
Total Hip Replacement	5	7.9
Internal fixation (Dynamic hip screw/Intra-medullary nail)	27	42.9
Person taking the consent		
Consultant	1	1.6
Registrar	29	46.0
Senior House Officer/Core Trainee	33	52.4

The main aim of our study was to determine the completeness of the documentation of the risks associated with the surgery. This has been summarized in Table 2. The most frequently documented risks were infection, blood clots (deep vein thrombosis and pulmonary embolism), and bleeding, with documentation rates of 98.4%, 95.2%, and 92.1%, respectively. Prosthetic joint dislocation following hemiarthroplasty or total hip replacement was not mentioned in 22.2% of the forms. Neurovascular injury was not documented in 20.6% of the consent forms. Less than 75% documentation rates were observed for postoperative pain (74.6%), anaesthetic complications (73%), failure (malunion/non-union/loosening of prosthesis) (68.3%), leg length discrepancy (60.3%), bone damage/fracture (50.8%), death (49.2%), wound-related complications/scars (42.9%), and hip stiffness (14.3%). None of the patients had been advised about the probable need for catheterization following surgery. It was worth noting that the intended benefits of the surgery (restoration of mobility and pain relief) were not documented in 12.7% (n=8) of the forms.

**Table 2:** Summary of the documentation rates of the risks associated with surgery

Risks	Documentation rate (%)
Infection	98.4
Blood clots (Deep vein thrombosis, pulmonary embolism)	95.2
Bleeding	92.1
Neuro- Vascular injury	79.4
Joint dislocation	77.8
Post operative pain	74.6
Anaesthetic complications	73.0
Failure/malunion/non-union/loosening of prosthesis	68.3
Leg length discrepancy	60.3
Bone damage/fracture	50.8
Death	49.2
Wound related complications/scar	42.9
Hip stiffness	14.3
Catheterization	0

On further analysis of the consent forms, we noted that 28.6% (n=18) did not mention the responsible consultant for the patient. Patient identification details (name, date of birth, hospital number, and NHS number) were incomplete on only one consent form. The title of the procedure and the side of the surgery were correctly documented in all forms. However, the diagnosis was not mentioned in 22.2% (n=14) of the forms. The possible need for blood transfusion was not documented in 25.4% (n=16) of the forms. In addition, 23.8% (n=15) of the forms did not mention the type of anaesthesia. The consenting doctor’s details (name, title, signature, and date of signing) were complete in all but one consent form. Although all the consent forms were signed by the patients, 57.1% (n=36) of them did not have the patient’s name and date of signing adjacent to the signature (Table 3). 

**Table 3:** Summary of the completeness of the remaining components of the consent form

Component assessed	Documentation rate (%)
Title of the surgery	100
The side of the injury and surgery	100
Patient identification details (name, Date of birth, sex, hospital number)	98.4
Consenting doctor details (name, title, signature, and date)	98.4
Intended benefits	87.3
Diagnosis	77.8
Type of anaesthesia	76.2
Probable need for blood transfusion	74.6
Responsible Consultant name and job title	71.4
Patient signing area	
Signature	100
Patient name and date of signing	42.9

## Discussion

Hip fractures account for the highest morbidity and mortality among fragility fractures and incur the highest cost in terms of treatment and rehabilitation [7-9]. Most patients with hip fractures are over the age of 65 and have multiple co-morbidities, which contributes to their high mortality [10,11]. It has been projected that close to 100,000 neck and femur fracture surgeries will be carried out annually in England by the year 2033 [12]. It has been shown that the one-year mortality is 20% lower in those that are operated on within 48 hours [5,6]. Thus, in our orthopaedic unit, we tend to prioritize hip fractures on the trauma surgical list to ensure that this 48-hour time limit will not be breached. The downside of this is that patients do not get ample time to deliberate on the surgery and its risks and benefits. In addition to this most often, consents for these surgeries are obtained within the busy emergency unit on first contact with the orthopaedic team. Therefore, often, the discussion surrounding consent can be hurried, and thus, the patient may not get a full description of what the procedure entails and what risks may follow. In this audit, we hoped to assess the adequacy of the information provided through the evaluation of the completeness of the written consent forms for neck of femur fracture surgeries, with special emphasis on the documentation of risks associated with the procedure.

Both the GMC and RCS England guidance clearly outline that when obtaining consent, patients should also be informed of the diagnosis, the expected benefit, and the surgeon involved in the procedure [1,2]. However, we found that in our study, 22.2% (n=14) of the consent forms did not contain the diagnosis or the indication for surgery, 12.7% (n=8) did not mention the intended benefits, and 28.6% (n=18) of the consent forms had no mention of the responsible consultant. The use of stickers containing patient identification details ensured that all but one consent form had the correct patient details. A significant proportion of patients with hip fractures may require blood transfusions at some point, either before surgery or postoperatively. This can be due to pre-existing anaemia and/or intra-operative blood loss. The need for blood transfusion tends to be relatively greater for inter-trochanteric fractures as opposed to intra-capsular fractures [13-15]. However, in our study, we noted that in 25.4% (n=16) of the cases, the possible requirement of a blood transfusion had not been mentioned.

As outlined by the Supreme Court in the case of Montgomery vs. Lanarkshire Health Board in 2015, doctors are bound to advise patients about the inherent risks associated with procedures that any reasonable person would deem significant [1,2]. The model consent form for hip fracture surgeries that was obtained from the www.orthoconsent.com website classifies risks associated with surgery based on their relative probability in three categories. Common risks (2%-5%) were blood clots (deep vein thrombosis and pulmonary embolism), bleeding, pain, and joint dislocation. Less common risks (1%-2%) were infection, leg length discrepancy, and catheterization. Rare risks (<1%) were wound-related complications or scars, neuro-vascular injury, bone damage or fracture, hip stiffness, failure, and death. In our study, we also included anaesthetic complications, as this is not documented elsewhere. Of the common risks, bleeding and blood clots were mentioned in over 90% of the forms. However, postoperative pain and joint dislocation were not mentioned in over 20% of the forms. Under the less common risks, infection was mentioned in 98.4% of the forms, while the possibility of leg length discrepancy was mentioned only in 60.3%. The possibility of catheterization was not mentioned in any of them. The documentation of rare risks was poor in general, as bone damage or fracture, wound-related complications, hip stiffness, and death were not mentioned in 50% or more of the forms. Neuro-vascular injury and failure (malunion/non-union/loosening of prosthesis) were mentioned in 79.4% and 68.3%, respectively. Anaesthetic complications were mentioned in 73% (n=46) of the forms.

It was noted in our study that the operative risks that are non-specific and common to most surgeries (bleeding, infection, blood clots, postoperative pain, and neurovascular injury) were more likely to be documented as opposed to complications that were more specific to neck of femur fracture surgeries and other orthopaedic surgeries (leg length discrepancy, joint dislocation, bone damage or fracture, hip stiffness, and failure). This association was found to be statistically significant (p<0.00001) after being analyzed using the Chi-Squared test, with a p<0.05 taken as statistically significant. As the majority of the consents were taken by doctors with no formal training in orthopaedic surgery, the relative lack of orthopaedic knowledge and operative experience may have contributed to the above finding. A similar study on consenting for neck and femur fracture surgeries, conducted at Wythenshawe Hospital in Manchester, United Kingdom, showed comparable results. Their study too showed that the documentation rates of risks such as infection, blood clots, bleeding, and neurovascular injury were relatively higher compared to risks such as hip stiffness, leg shortening, and fracture [16]. Both GMC and RCS guidance state that the discussion with the patient should be carried out by the surgeon or someone delegated by the surgeon who is appropriately trained and competent to have a comprehensive discussion with the patient, covering the procedure, intended benefits, probable risks and complications, and alternate treatment options available [1,2]. As we only assessed the written consent forms in our audit, it is possible that some of the risks associated with the surgery may have been discussed with the patient but may not have been documented on the consent forms. This was identified as a limitation in our study.

## Conclusions

Our study revealed several deficiencies in the consenting of patients for neck and femur fracture surgeries. There were poor documentation rates for risks associated with surgery, especially the less common and rare ones. We also noted that risks that were more orthopaedic-oriented were less likely to be mentioned, as opposed to general risks that are common to most surgeries. This was found to be statistically significant. This may be attributed to the lack of adequate orthopaedic knowledge and experience among the senior house officers and core trainees, who were most often responsible for consenting. We also noted several deficiencies in the completion of the consent forms that were not in keeping with the GMC and RCS guidance on consent.

As senior house officers and core trainees don't often have formal orthopaedic training, they may not have adequate orthopaedic experience or knowledge to carry out a good discussion with the patient and consent subsequently. Therefore, we recommend that at the induction of each batch of new senior house officers and core trainees, a teaching session be carried out to advise on good consenting practices and to improve their knowledge on hip fractures, their surgical management, and the probable risks. We also presented the above findings at the orthopaedic clinical governance meeting to raise awareness about the deficiencies within the department. We also recommend printing out the model hip fracture consent form on the www.orthoconsent.com website and putting it up on the trauma room notice board so that junior doctors can see it regularly and refresh their knowledge. We recommend re-auditing the study after a new batch of doctors arrives to ensure that these changes have brought about the desired effect.
